# Integrated Management of Type 2 Diabetes and Gestational Diabetes in the Context of Multi-Morbidity in Africa: A Systematic Review

**DOI:** 10.5334/ijic.5608

**Published:** 2022-09-21

**Authors:** Jean Claude Mutabazi, Mahmoud Werfalli, Angeli Rawat, Ezekiel Musa, Tawanda Chivese, Shane Norris, Katherine Murphy, Helen Trottier, Naomi Levitt, Christina Zarowsky

**Affiliations:** 1 Département de Médecine Sociale et Préventive, École de Santé Publique, Université de Montréal, Pavillon 7101, Avenue du Parc, Montreal, QC, H3N 1X7, Canada; 2 Centre de Recherche en Santé Publique (CReSP), Université de Montréal et CIUSSS du Centre-Sud-de-l’Île-de-Montréal, Montréal, Canada; 3 Centre de Recherche du Centre Hospitalier Universitaire Sainte Justine, Montréal, H3T 1C5, QC, Canada; 4 Department of Medicine, Faculty of Health Science, University of Cape Town, Chronic Disease Initiative for Africa, Cape Town, Western Cape, South Africa; 5 The School of Population and Public Health, University of British Colombia, Vancouver, Canada; 6 Integrated Intervention for DIAbetes risks after GestatiOnal diabetes (IINDIAGO), Department of Medicine, Faculty of Health Science, University of Cape Town, Cape Town, Western Cape, South Africa; 7 University of Witwatersrand, Paediatrics and Child Health Johannesburg, Gauteng, South Africa

**Keywords:** integrated care, diabetes mellitus, type 2 diabetes, gestational diabetes, multimorbidity, syndemic, health systems

## Abstract

**Introduction:**

Many adults diagnosed with gestational diabetes mellitus (GDM) and type 2 diabetes mellitus (T2DM) also have other known or unknown comorbid conditions. The rising prevalence of GDM and T2DM within a broader context of multimorbidity can best be addressed through an integrated management response, instead of stand-alone programs targeting specific infectious and/or chronic diseases.

**Aim:**

To describe GDM and T2DM screening, care and cost-effectiveness outcomes in the context of multimorbidity through integrated interventions in Africa.

**Methods:**

A systematic review of all published studies was conducted according to the Preferred Reporting Items for Systematic Reviews and Meta-Analyses (PRISMA) guidelines. Risk Of Bias in Non-randomised Studies of Interventions (ROBINS-I) was used to assess risk of bias. Data synthesis was conducted using narrative synthesis of included studies.

**Results:**

A total of 9 out of 13 included studies reported integrated diabetes mellitus (DM) screening, 7 included integrated care and 9 studies addressed cases of newly detected DM who were asymptomatic in pre-diabetes stage. Only 1 study clearly analysed cost-effectiveness in home-based care; another 5 did not evaluate cost-effectiveness but discussed potential cost benefits of an integrated approach to DM screening and care. Compared to partial integration, only 2 fully integrated interventions yielded tangible results regarding DM screening, care and early detection of cases despite many that reported barriers to its sustainability.

**Conclusion:**

Though few, integrated interventions for screening and/or care of DM in the context of multimorbidity within available resources in health systems throughout Africa exist and suggest that this approach is possible and could improve health outcomes.

## Background

Gestational Diabetes Mellitus (GDM) and Type 2 Diabetes Mellitus (T2DM) are the two types of diabetes commonly identified during adulthood and comprise more than 90% of global diabetes cases [1,2]. Their prevalence has been rising worldwide, especially in the context of multiple co-morbidities and risk factors in LMICs, despite the history of underdiagnosis and low reporting in these countries [3,4,5,6,7,8,9]. Several factors contribute to this increasing burden. First, some women with T2DM are diagnosed for the first time during their pregnancy and are included among women with GDM [10]. Women with true GDM and not previously undiagnosed T2DM are at high risk of developingT2DM in the long term, and their children are also at risk [10,11]. Secondly, triggered by genetic and environmental factors through epigenetic mechanisms [12], both GDM and T2DM occur later in life, in a population that increasingly becomes vulnerable to various other risk factors and complications [11,13,14,15]. Thirdly, adults diagnosed with GDM or T2DM may remain unaware that they have diabetes and may also suffer from other known or unknown comorbid conditions. Chronic co-morbid conditions could include cardio-vascular diseases (e.g., hypertension) and/or infectious diseases (e.g., tuberculosis, Hepatitis B, HIV/AIDS) and/or vector borne diseases (e.g., malaria) [16]. Treatment of some of these diseases – such as antiretroviral therapy (ART) for HIV – may increase the likelihood of concomitant metabolic complications, with possible pre-existing opportunistic infections among others [10,13,17,18,19,20,21,22,23,24,25,26,27,28], necessitating more complex and costly clinical management [16]. The multimorbidity caused by comorbid non-communicable and infectious chronic diseases [29], which include GDM and T2DM, has not been well studied, especially their integrated management into primary health care (PHC) in LMICs including all countries in Africa. Fourthly, the diagnosis of GDM or T2DM among some patients with multiple diseases has to be conducted along with diagnosis of these other multiple diseases, a situation that causes challenges in terms of cost and logistics for adequate testing and management, especially in the context of struggling health systems. Hence, researchers and experts increasingly argue that the rising prevalence and burden of GDM and T2DM [1,30,31], can best be addressed through an integrated management response instead of more easily delivered and less costly stand-alone programmes targeting specific diseases [29,32,33,34,35].

Syndemic theory is increasingly used as a framework not only to understand but also to also design interventions for complex multiple diseases affecting disadvantaged populations, especially in low- and middle-income countries (LMICs). LMICs in Africa and beyond are facing epidemiological transitions [36,37], that overwhelm already weak health systems dealing with multiple complex health problems rather than a single disease or isolated risk factors [38]. A syndemic framework assesses and addresses interacting population health problems where underlying biological, cultural, socioeconomic and environmental dimensions lead to health inequities [37,39]. It also goes beyond conventional approaches to co-morbidity and multimorbidity [40] such as disease-specific, stand-alone or vertical programmes for targeted infectious, non-communicable, acute or chronic conditions and specific co-morbidities. It instead suggests that integrated management of multiple conditions, though not simple, is necessary for better health services delivery [41]. In contrast to documented integrated interventions within multimorbidity in the developed world, findings from LMICs are scarce, especially in the context of colliding infectious and chronic diseases including GDM and T2DM in LMICs, especially in Africa [29,42,43,44,45,46].

This study aimed to review the literature on integrated management of T2DM and GDM in the context of multimorbidity in Africa and to identify the emerging good practices, lessons and advantages, including cost-effectiveness, of integrated rather than vertical or targeted interventions. Additionally, we identified research gaps related to GDM and T2DM integration within management of other chronic and infectious diseases and propose syndemic theory as a useful conceptual background to this study.

This systematic review answers the following research questions: 1) What are the existing integrated interventions and service delivery models for managing T2DM including GDM in the context of multi-morbidity in Africa? 2) What are the successes and challenges of the existing integrated management of T2DM including GDM in the context of multi-morbidity in Africa?

## Methods

### Protocol

The protocol for this study was developed based on the Cochrane Handbook for Systematic Reviews [47] and registered with PROSPERO: (https://www.crd.york.ac.uk/prospero/), registration no. CRD42016046630. The systematic review methods were described in our previously published protocol [48].

### Study design and search strategy

For this study, the PRISMA guideline was followed during the systematic review [49]. Published studies were searched using terms (MeSH: Medical subject heading) and key words. The following databases were searched: Cochrane Library, MEDLINE, PubMed, Embase, SCOPUS, AIDS journal and the Cumulative Index to Nursing and Allied Health Literature (CINAHL). Additionally, a manual search was conducted in Google scholar, ClinicalTrials.gov (ClinicalTrials.gov) *and relevant journals for additional studies*. The target population, the intervention of interest, the comparator intervention, key outcomes and time (PICOT) approach [50] was used as a framework for the identification and selection of studies for inclusion. This study was limited to all fifty-four African countries. Since there were not many articles regarding our review topic in our preliminary search, there were no starting time limits up to the search date in February 2019 but two full papers published later in 2019 were extracted after their conference abstracts were initially included in the selection. The search strategy used is shown in Table 1.

**Table 1:** Search strategy.


(integrat* OR linkag* OR combin* OR amalgamat* OR coordinat* OR unificat* OR manag* OR comprehensive* OR “co-ordinated” OR “disease control” OR care deliver* OR “healthcare deliver*” OR “health care deliver*” OR “collaborative care” OR “intersectional collaborat*” OR “interagency collaborat*” OR “care partner*”)	AND	(diabet* OR diabetes mellitus, type 2/OR diabetes, gestational/)	AND	(comorbid* OR co-morbid* OR multimorbid* OR multi-morbid* OR polymorbid* OR poly-morbid* OR codisease* OR co-disease* OR multidisease* OR multi-disease* OR polydisease* OR poly-disease* OR coillness* OR co-illness* OR multiillness* OR multi-illness* OR polyillness* OR poly-illness* OR copatholog* OR co-patholog* OR multipatholog* OR multi-patholog* OR polypatholog* OR poly-patholog* OR codisorder* OR co-disorder* OR multidisorder* OR multi-disorder* OR polydisorder* OR poly-disorder* OR cocondition* OR co-condition* OR multicondition* OR multi-condition* OR polycondition* OR poly-condition* OR cosyndrom* OR co-syndrom* OR multisyndrom* OR multi-syndrom* OR polysyndrom* OR poly-syndrom* OR ((coexisting OR co-existing OR multiple) W0 (morbidit* OR disease* OR illness* OR patholog* OR disorder* OR condition* OR syndrom*)) OR ((Charlson* OR Elixhauser*) W0 (index* OR score*))) OR OR “noncommunicable disease*” OR “non communicable disease*” OR ncd OR ncds OR “non infectious disease*” OR “non infectious illness*” OR “chronic disease*” OR “chronic illness*” OR “cardiovascular disease*” OR “vascular disease*” OR “heart disease*” OR “heart illness*” OR “cardiac disease*” OR “heart attack*” OR stroke* OR “heart failure” OR “heart rupture” OR “cardiac arrest” OR cancer* OR neoplasm* OR “chronic respiratory disease*” OR “chronic airflow obstruction*” OR “chronic obstructive airway disease*” OR “chronic obstructive lung disease*” OR “chronic obstructed pulmonary disease*” OR asthma OR “lung disease*” OR “communicable disease*” OR “infectious disease*” OR “human immunodeficiency virus” OR hiv OR “acquired immunodeficiency syndrome” OR aids OR “opportunistic infectious disease*” OR tuberculosis OR tb OR malaria OR pneumonia OR “diarrheal disease*”)	AND	Africa/OR “Africa South of the Sahara”/OR “Sub-Saharan Africa”/OR north Africa/OR Africa, Northern/Egypt or Libya OR Tunisia OR Algeria OR Morocco OR “Western Sahara” OR Angola/OR Benin/OR Botswana/OR Burkina Faso/OR Burundi/OR Cameroon/OR Cape Verde/OR Central African Republic/OR Chad/OR Comoros/OR Congo/OR Brazzaville/OR Cote d’Ivoire/OR Djibouti/OR Equatorial Guinea/OR Eritrea/OR Ethiopia/OR Gabon/OR Gambia/OR Ghana/OR Guinea/OR Bissau/OR Kenya/OR Lesotho/OR Liberia/OR Madagascar/OR Malawi/OR Mali/OR Mauritania/OR Mauritius/OR Mozambique/OR Namibia/OR Niger/OR Nigeria/OR Rwanda/OR Sao Tome e Principe/OR Senegal/OR Seychelles/OR Sierra Leone/OR Somalia/OR South Africa/OR South Sudan/OR Sudan/OR Swaziland/OR Eswatini OR Tanzania/OR Togo/OR Uganda/OR Western Sahara/OR Zaire/OR Zambia/OR Zimbabwe/


### Study selection

After systematic searches, the retrieved citations were exported to and managed using Endnote X9. Duplicates were removed automatically and a manual search was conducted to crosscheck and remove any duplicates that escaped the automatic removal. The remaining citations were independently screened for eligibility by two researchers (JCM and EM), in accordance with the inclusion and exclusion criteria of the study. Any disagreements were resolved through discussion between reviewers and with a third author (TC). These citations were assessed in two phases by two researchers (JCM and EM); the titles and abstracts first and then the full-text articles of potential studies for inclusion. Once titles and abstracts were screened, the full text were retrieved and screened for eligibility. The team of three researchers (JCM, EM and TC) discussed and agreed on the final studies included.

### Eligibility criteria

Eligible studies were the published and unpublished randomised controlled trials (RCTs), non-RCTs, quasi-randomised controlled trials (QCTs) and observational studies on integrated interventions for management of T2DM and GDM within multi-morbidity conditions in Africa, without language restrictions. Studies that were considered for inclusion were primarily quantitative but also included a limited number of relevant qualitative and mixed methods studies. Because most of the included studies simply used “diabetes mellitus” (DM) as a classification instead of the standardised classification of type 1, type 2, GDM or other specific types of diabetes [51,52] in the context of multimorbidity, we considered DM instead of T2DM and GDM. To ensure that the DM discussed was either GDM or T2DM and therefore eligible for inclusion (for the reasons explained in the Introduction, above), we first checked whether the screening and/or care of DM or its early case detection were among adult patients without a pre-existing diagnosis of type 1 diabetes. All screenings and subsequent procedures were indicated as conducted for the first time without prior diagnosis which increased our confidence that they would in fact be GDM or T2DM, if standard classifications were applied. The level of integration of the intervention [53,54] was considered. Included interventions could be: (1) mainstreamed (disease specific programmes that were included into PHC services), (2) partially integrated (through linkage or unstructured interactions of two or more disease-specific programmes and possibly including the coordination of interactions with a committee to oversee work oriented to shared goals but maintaining separate programmatic and administrative structures) or (3) fully integrated (in which two or more disease specific programmes were structurally merged including funds, human resources, information system and functional elements such as strategic planning, resource allocation, intervention delivery)(53,54). The outcomes within the multimorbidity framework that were considered in these integrated interventions with these chronic diseases were: integrated screening, integrated care (preventive, treatment and referral services), cost-effectiveness, and early detection of disease.

### Data extraction

Data were extracted using a piloted form. The following information was extracted for each included study: the characteristics of the eligible research reports (author(s), year of publication, country of study, and study setting); study methods (study design, target population, sampling strategy, total number of participants, and response rate); intervention and facility (diagnosis, other co-morbidities, service providers (Doctor, Nurse, Both), and point of entry/type of facility); study outcomes (integrated screening outcome, integrated care outcome (preventive, treatment and referral services), cost-effectiveness outcome, and early detection of disease outcome); and approach and level of integration (integration through co-location of services (same room or same clinic), integration of two services OR integration into PHC-mainstreaming, partial integration (linkages, coordination), and full integration.

### Assessment of risk of bias in included studies

MW and JCM assessed the methodological quality and risk of bias of the included studies using the ROBINS-I assessment tool [55].

### Data synthesis

We performed a narrative synthesis [56], to summarize and thoroughly compare a variety of included studies. We then presented findings through different outcomes and a tabular summary was used to synthesize individual studies characteristics and outcomes (intervention effects). Heterogeneity of the populations as well as of the included studies made a meta-analysis inappropriate for this systematic review.

### Patient and public involvement

No patients were involved in the development of the research question, the design or the conduct of this study.

## Results

### Description of included studies

**Figure 1:**
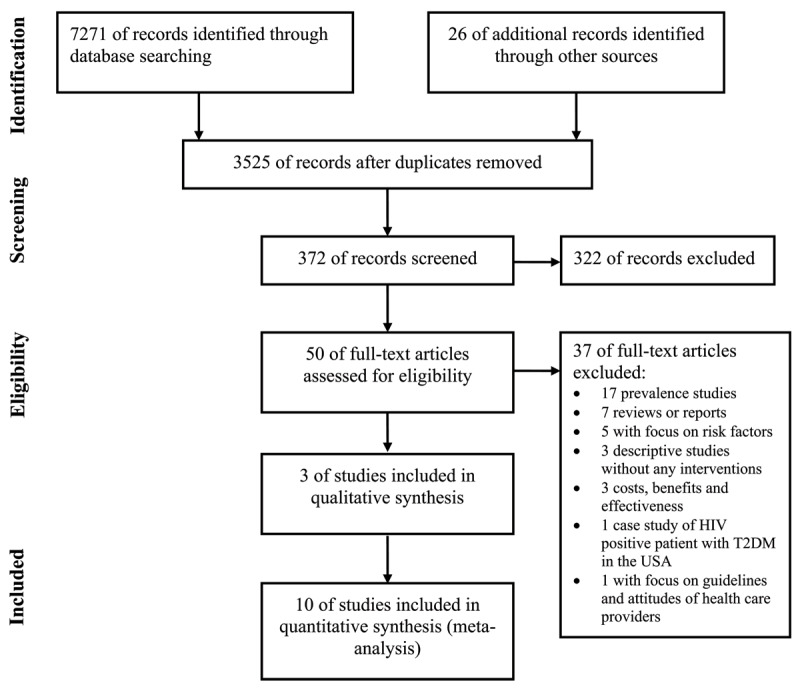
Flow diagram of the included studies for the systematic review of integrated management of type 2 diabetes mellitus and gestational diabetes mellitus in the context of multi-morbidity conditions in Africa.

A total of 7297 published articles were retrieved; 3772 duplicate records were removed and 3153 records were excluded after screening title and abstract. A total of 372 full-text articles were screened for eligibility. Of those, 322 full-text articles were excluded, because they failed to fulfill prior eligibility criteria and out of 50 potential studies 37 articles were excluded for cited reasons. Finally, 13 studies were included in the final analysis, 3 for narrative synthesis and 10 for quantitative analysis (Figure 1).

### Characteristics of the included studies

All the included studies were from only seven African countries: eight were from Southern African countries – four from South Africa [57,58,59,60], three from Malawi [61,62,63] and one from Angola [64], four were from East African countries; two from Kenya [65,66], one from Ethiopia [67], one from Uganda [68] and one from Central Africa (Cameroon) [69]. Regarding the study design, most studies (10/13) were cross-sectional [57,59,60,61,62,63,64,67,68] and three were cohort studies [58,65,69]. A total of 27,772 participants were included in this review and some were purposively sampled [61,63,64,66,67,69] while others were on voluntary [58,62,65], random [59], quota from an old enrolled cohort [60], convenience [57] and community based campaign [68] sampling bases. Table 2 details the characteristics of the included studies.

**Table 2:** Study characteristics.


AUTHOR	YEAR	COUNTRY	STUDY AIM	STUDY DESIGN	SAMPLING STRATEGY

Labhardt N.D et al.	2010	Cameroon	To examine the effectiveness of integrating care for HT and T2DM by task shifting to non-physician clinician (NPC) facilities in eight rural health districts in Cameroon.	Cohort	Purposive

Segafredo G. et al.	2016	Angola	To estimate the double burden of DM, HT and TB and to pilot the integration of the screening for DM and HT in the TB national programs in six TB centers in Luanda.	Cross-sectional or Interventional	Purposive

Wroe E.B. et al.	2015	Malawi	To increase access to care for NCD patients, to maximize efficiency given the severe human resource shortages, and to replicate strong HIV outcomes for patients with other chronic conditions.	Cross-sectional	Purposive

Chamie G.et al.	2012	Uganda	To test the feasibility and diagnostic yield of integrating NCD and other communicable disease services into a rapid, high-throughput, community-based HIV testing and referral campaign for all residents of a rural Ugandan parish, and to determine rates and predictors of post-campaign linkage to care by disease.	Cross-sectional	Community based campaign (census)

Jerene D. et al. 2017 Ethiopia	2017	Ethiopia	To demonstrate the feasibility of integrated care for TB, HIV and DM in a pilot project.	Cross-sectional	Purposive

Almossawi HJ et al.	2019	South Africa	To assess the readiness of the PHC system to provide integrated TB and DM services.	Cross-sectional	Random for patients records, convenience for respondents

Pfaff C. et al. 2018 Malawi	2018	Malawi	To describe the experience of this pilot initiative, where all adults accessing care in the HIV clinic are screened and treated for HT and DM during the same visit.	Cross-sectional	Purposive

Pastakia S.D et al.	2017	Kenya	To assess the impact of the implementation of a patient-centered rural NCD care delivery model called Bridging Income Generation through grouP Integrated Care (BIGPIC).	Cohort or Interventional	Voluntary

Govindasamy D. et al. 2013	2013	South Africa	To determine the yield of newly-diagnosed HIV, TB symptoms, DM and HT, and to assess CD4 count testing, linkage to care as well as correlates of linkage and barriers to care from a mobile testing unit.	Cohort or Interventional	Voluntary

Kachimanga C. et al.	2017	Malawi	To increase case detection for NCDs in the community, at the facility for acute outpatient care, and at Integrated Chronic Care Clinic (IC3) itself.	Cross-sectional	Voluntary

Manne-Goehler J. et al.	2017	South Africa	To assess the relationship between ART use and utilization of health care services for DM and HT.	Cross-sectional	Random

Golovaty I. et al.	2018	South Africa	To conduct a cost analysis to determine the per-person incremental costs associated with integrating NCD screening and counseling to a home-based HIV counseling and testing program in KwaZulu-Natal.	Cross-sectional	Quota from an old enrolled cohort

Venables A. et al.	2016	Kenya	To assess patient and health-care worker perceptions and experiences of medicines adherence clubs (MACs) in the urban informal settlement of Kibera.	Cross-sectional	Purposive


### Quality appraisal and risk of bias for included studies

All studies reported the results of nonrandomized studies. Many biases were recorded while analyzing all studies and only one study was considered to have a low risk [69] while the remaining twelve had serious risk of bias [57,58,59,60,61,62,63,64,65,66,67,68]. Studies with serious risk of bias either lacked or had unclear information on participant selection, classification of interventions, measurement of outcomes, selection of the reported results. Some biases were caused by confounding, deviation from intended interventions or by missing data (Table 3).

**Table 3:** Results of the assessment of risk of bias in included studies by using the ROBINS-I assessment tool.


STUDY ID	1. BIAS CAUSED BY CONFOUNDING	2. BIAS CAUSED BY SELECTION OF PARTICIPANTS	3. BIAS CAUSED BY CLASSIFICATION OF INTERVENTIONS	4. BIAS CAUSED BY DEVIATIONS FROM INTENDED INTERVENTIONS	5. ATTRITION BIAS CAUSED BY MISSING DATA	6. DETECTION BIAS CAUSED BY MEASUREMENT OF OUTCOMES	7. REPORTING BIAS CAUSED BY SELECTION OF THE REPORTED RESULTS	OVERALL JUDGEMENT

Labhardt N.D et al. 2010 Cameroon	Low	Low	Low	No information	Low information on reasons for missing data provided)	Low	Low	Low

Segafredo G. et al. 2016 Angola	Serious	Low	No information	No information	No information	Serious	No information	Serious

Wroe E.B. et al. 2015 Malawi	Serious	Serious	No information	No information	Serious	Serious	Serious	Serious

Chamie G.et al. 2012 Uganda	Serious	Serious	No information	No information	Serious	No information	No information	Serious

Jerene D. et al. 2017 Ethiopia	Serious	Serious	No information	No information	Low (information on reasons for missing data provided)	No information	No information	Serious

Almossawi HJ et al. 2019 South Africa	Serious	Low	No information	No information	Low (information on reasons for missing data provided)	No information	Low	Serious

Pfaff C. et al. 2018 Malawi	Serious	Low	Low	No information	Low (information on reasons for missing data provided	No information	No information	Serious

Pastakia S.D et al. 2017 Kenya	Serious	Serious	Serious	Low	No information	Serious	Serious	Serious

Govindasamy D. et al. 2013 South Africa	Low	Low	No information	No information	No information	No information	No information	Serious

Kachimanga C. et al. 2017 Malawi	Serious	Serious	No information	No information	No information	No information	No information	Serious

Manne-Goehler J. et al. 2017 South Africa	Serious	Low	No information	No information	Low (information on reasons for missing data provided	Serious	No information	Serious

Golovaty I. et al. 2018 South Africa	Low	Low	No information	No information	No information	No information	No information	Serious

Venables A. et al. 2016 Kenya	Serious	Serious	No information	No information	Serious	No information	No information	Serious


### Outcomes from included studies

Included studies have shown that integrated screening and care of DM as well as the early detection of DM cases in the context of multimorbidity is possible, although these studies were few and had significant heterogeneity in their findings. Outcomes regarding the integrated screening, care and early detection of DM cases, as well as the cost-effectiveness and integration level outcomes from the included studies were analyzed, summarized and were subsequently presented in Table 4, under the following themes:

**Table 4:** Cost effectiveness outcomes and integration levels.


AUTHOR	YEAR	COUNTRY	COST-EFFECTIVENESS OUTCOME	INTEGRATION LEVEL: PARTIAL*/FULL**

Labhardt et al.	2010	Cameroon	Affordable drugs from the national essential drug list were available and used	Full

Segafredo et al. 2016	2016	Angola	Not measured	Partial

Wroe et al.	2015	Malawi	All patients were seen in one day, at the nearest health center, for all of their chronic conditions.	Full

Chamie G.et al.	2012	Uganda	Cost-effectiveness of adding NCD screening was not the aim of the study but the relatively low cost of $2.41/person makes it likely to be cost-effective.	Partial

Jerene et al.	2017	Ethiopia	Not measured	Partial

Almossawi et al.	2019	South Africa	Not measured	Partial

Pfaff et al.	2018	Malawi	Not measured but its advantages discussed and its evaluation recommended	Partial

Pastakia et al.	2017	Kenya	group care model resulted in 72.4% of screen-positive participants returning for subsequent care, of which 70.3% remained in care through the 12 months of the evaluation period.	Partial

Govindasamy et al.	2013	South Africa	Not measured	Partial

Kachimanga et al.	2017	Malawi	Not measured	Partial

Manne-Goehler et al.	2017	South Africa	Not measured	Partial

Golovaty et al.	2018	South Africa	Comprehensive home-based HIV-NCD testing and counseling results in a modest increase in costs with the potential to avert NCD death and disability. The additional time burden of NCD screening and testing was the major driver of costs, emphasizing the need for a targeted approach that bridges to an integrated public health model. 20% increase in testing and counseling time was revealed in time assessment	Partial

Venables et al.	2016	Kenya	MACs allow for the efficient management of co-morbidities and enable large numbers of stable patients to collect their chronic medication efficiently, whilst simultaneously enabling patients to benefit from peer support and health education.	Partial


Partial *: Integration through co-location of services (same room or same clinic). Full **: Integration of services OR integration into PHC-mainstreaming.

#### Integrated diabetes mellitus screening and care in Africa

One included study clearly mentioned that the type of diabetes was T2DM [69], while the remaining studies used DM as diabetes diagnosed or treated along with other diseases from which patients suffered. Among twelve studies that reported DM screening among other co-morbidities [57,58,59,60,61,62,63,64,65,67,68,69], only nine reported the exact number of patients who were screened. As expected, the review found that different criteria were used to diagnose DM in routine screening. Applying different criteria was an additional challenge in Africa where lack of clear protocols, limited resources in health facilities and inadequate training for health workers, especially at primary levels of care, was widely documented [57,61,69]. Though integrated DM screening was identified in most retained studies, not all patients who benefited from integrated screening had integrated treatment.

Integrated care including preventive, treatment and referral services was reported in nine studies [57,58,59,61,62,63,66,68,69], out of which seven had a known number of patients in care. DM screening in the reviewed studies was conducted at different venues: four at the clinic or PHC facilities exclusively [61,63,66,69], three at home or community based infrastructures [60,65,68], five at specialized clinics or clinics in close collaboration with hospitals or at hospitals [57,59,62,64,67] and one at a mobile clinic [58], and by different teams. Health care workers involved in screening and care of DM within multi-morbidities ranged from expert clients or trained patients [63] and lay counsellors and community health workers [58,59,60,61,62,65,67] playing limited roles, to nurses and clinicians that lead interventions in all 13 included studies. The expertise and available resources, including equipment and medication in the facilities, were highlighted as key factors for the successful implementation of integrated screening and care of DM and other NCDs. This integration was more easily carried-out when conducted within the existing protocols of well-established programmes such as for HIV and these established programmes were seen as of tremendous impact to its success [59,61,62,63]. In fact, nine integrated DM screening and care interventions included in this study were conducted with HIV as one of the multi-morbidities [58,59,60,61,62,63,66,67,68]. Tuberculosis [57,58,62,64,67,68], malaria [68], hypertension [59,63,65,69] and other NCDs including, depression, cardiovascular disease, and health risks such as tobacco, obesity and alcohol use [58,60,61,62] were other diseases and risk factors screened or treated along with DM.

#### Early detection of DM cases

Nine studies addressed the cases of newly detected DM who were asymptomatic and those with impaired glucose or in pre-diabetes stage [58,61,62,63,64,65,67,68,69]. The remaining four studies did not measure this outcome nor include it as one of its results [57,59,60,66]. One study was not included in the meta-analysis as it only mentioned this particular outcome in their weekly integrated screening at the clinic and during outreach but did not share the number of early detected DM cases [61].

#### Cost-effectiveness of integrated DM screening and care

The majority of included studies (7/13) did not evaluate cost-effectiveness [57,58,59,62,63,64,67]. Only one study clearly analysed cost-effectiveness of home-based integrated screening and referral to care of HIV and comprehensive NCDs including DM [60]; another five did not evaluate cost-effectiveness but rather discussed potential cost benefits of an integrated approach to DM screening and care [21,61,65,66,69]. Some of the elements addressed throughout different studies that were highlighted and that could relate to cost-effectiveness were: patients with multi-morbidities being seen in one day for all their health conditions [61], availability and affordability of essential DM/NCDs drugs [68,69], efficient collection of DM and other NCDs medication, benefiting from peer support and health education [66] and reinforcement of adherence to care [65].

#### Integration levels for GM screening and care within multimorbidity

Interventions carried out in the included studies were integrated but at different levels based on the study objectives, design or available resources for services delivery. Only two studies were classified as fully integrated but not mainstreamed (i.e., services offered for two or more diseases were merged in structural and functional aspects but were not delivered along with other primary care services). Wroe et al. in Malawi and Labhardt et al. in Cameroon reported services that were fully integrated [61,69] and provided DM screening and care following a clear protocol within the package of other services available in the health care facility. The other 11 studies were partially integrated [57,58,59,60,62,63,64,65,66,67,68], which means that the services were offered through coordination or co-location in the same room or same clinic but each programme kept its structures as separate entities within health care services.

### Integration approaches and models of DM screening and care

Most studies included in this review did not apply specific approaches or models to integrate DM screening and care in the context of multimorbidity. However, some details emerged from a small number of reviewed studies that gave limited information regarding intervention approaches or models used to achieve the aimed integration of screening or care of DM. Task shifting to non-physician clinicians [69], the integrated Chronic Care Clinic, locally called IC3 or “Ice-Cubed” through task shifting and decentralisation [61,62], medication adherence clubs [66], mobile testing [58] were the few documented approaches adopted to integrate screening or care DM in the context of multimorbidity. Other studies strived for integration of screening or managing DM along with other services or available protocols in the facility but without a specified model used for this particular purpose.

## Discussion

This was a systematic review that examined: 1) the existing integrated interventions and service delivery models for managing T2DM including GDM in the context of multi-morbidity in Africa; and 2) the successes and challenges of the existing integrated management of T2DM including GDM in the context of multi-morbidity in Africa.

In most high-income countries, patients with multi-morbidities including NCDs like DM have been documented to have access to family doctors or general practitioners and health care facilities equipped to provide appropriate integrated care and address multiple health problems [35,70,71,72]. In contrast, Africa does not generally possess enough facilities and the required resources to offer integrated care models like the Integrated Chronic Disease management (ICDM) model, Innovative Care for Chronic Conditions (ICCC) framework, among others [29,73,74,75] for DM. In the context of multimorbidity and severe resource constraints, few studies included in our review followed well-described integrated care models, as seen in the results. This highlights the urgent need to identify core indicators of integrated care models to allow for comparability and share lessons learned, which is increasingly important as health systems are tasked with caring for multimorbidities in the face of waning resources.

Only one study [69] by Labhardt et al. on the integrated intervention of hypertension and T2DM into PHC clinics conducted by clinical nurses in rural Cameroon (2010) assessed after two years, had a low risk of bias. We did not identify any RCTs.

The Labhart et al. study [69] highlighted that fully integrated management of DM is feasible. The findings demonstrate that with adequate training and supervision for nurses on T2DM prevention, diagnosis and care and the provision of additional needed equipment and drugs to the existing facilities within national health system framework, successful integration into PHC is possible. Another study conducted under the fully integrated chronic care clinic in Malawi by Wroe et al. [61] in 2015 had similar results. With lessons from a previously failed partial integration intervention, existing HIV platforms were used to benefit NCDs including DM in terms of prevention, diagnosis, care and follow-up to trace the defaulters [61]. Both of these fully integrated interventions have shown how tasks to prevent, screen and treat DM and other NCDs could be shifted from doctors to nurses and other health care workers in the clinics and communities. Drawing on the experience of scaling up HIV testing and care in Africa, task-shifting could be seen as a good strategy to increase the availability and accessibility of clinical services that are also cost-effective to deal with the rising burden of DM and other major NCDs at primary care [76,77,78,79,80,81]. Other studies included in this review were of partially integrated interventions that did not assess task shifting or task sharing aspect of services integration and were limited to either DM screening, care or both and other components as above shown in the results section. The main finding in relation to our study question was that fully integrated screening and care have been shown to work well within multimorbidity approaches in PHC, although only two studies covered this.

As one the main review outcomes, integrated DM screening conducted has led to early detection of unclassified DM in nine studies, and DM would be T2DM and GDM if it were categorised well in those respective studies. These newly screened patients were asymptomatic when diagnosed for the first time in the integrated package of services and had an opportunity to be initiated on treatment before complications appear, while those found to be in pre-diabetes stage with impaired glucose had time to change their lifestyle in order to prevent or delay DM onset [52,82,83]. While arguing that the early detection of T2DM should align with changes in LMICs’ health systems, Narayan et al. recommend an integrated approach to address the rapidly increasing T2DM rates and its associated complications or other NCDs in the most cost-effective way [84].

The last outcome from this review was cost-effectiveness, mentioned in seven studies. Only one study conducted by Golovaty et al. in South Africa in 2018 analysed the cost of the home-based integrated screening of NCDs including DM into HIV testing and counselling [60]. Others neither systematically measured the costs, nor the health outcomes [85] of DM integration as an intervention option within multimorbidity. Desmedt et al. in 2016 did not find any study from African country to include in their research assessing the economic impact of integrated care for patients with NCDs including T2DM [86]. The study by Pfaff et al. in 2018 included in this review did not find any publications with formal cost-effectiveness analysis of integrated management of NCDs and HIV [63]. HIV programs, especially in Africa could present an opportunity for measured integration of NCDs, against the potential cost-savings of integrated NCD screening and treatment [63,87,88].

## Strengths and Limitations

### Strengths

For this systematic review and meta-analysis, many databases were searched and all identified evidence of integrated management of T2DM and GDM within multimorbidity through-out the continent were analyzed, even though few studies qualified for inclusion. To our current knowledge, no other study has comprehensively assessed integrated management of DM within multimorbidity in Africa.

### Limitations

Many studies that could have enriched this review did not have the integrated DM screening and care interventions but merely focused on prevalence or others aspects that did not meet this study’s inclusion criteria. Lack of RCTs to meet the inclusion criteria reveals paucity of rigorous data and highlights the need for more research in this important health systems domain. The lack of studies from many sub-regions and countries in the continent may limit the generalizability of the findings. GDM as a specific health problem for a particular group of population prone to other diseases or risk factors did not clearly appear in studies included in this review and it would be important to consider it for further integrated services. Integrated care being itself a complex approach, most of included studies did not give information about the levels of integration and they were then classified based on the predefined research terms. With this challenge, it was also obvious that a study could set out to be one type of integration and then whether or not in reality that happens the way that was planned, was the next consideration. Another limitation involved heterogenous study designs, methods and outcomes of included studies which weaken the conclusions of the present study.

## Conclusions

All included studies demonstrated the feasibility and benefits of integrated management of DM within multimorbidity and emphasized the importance of integration in Africa. Only two studies reported on fully integrated interventions and both were successful. Some studies suggested that integrated interventions to screen and care for DM in the context of multimorbidity could potentially be cost-effective, although scarce evidence of its formal analysis was noted. More original research and review studies are needed to analyze integrated management of T2DM and GDM practices in the context of multimorbidity in Africa.

## Abbreviations

AIDS: Acquired Immunodeficiency Syndrome

ANC: Antenatal Care

ART: Antiretroviral Therapy

CCRBT: Cochrane Collaboration Risk of Bias Tool

CI: Confidence Interval

CNCICDs: Comorbid Non-Communicable and Infectious Chronic Diseases

DM: Diabetes Mellitus

EPHPP: Effective Public Health Practice Project

GDM: Gestational Diabetes Mellitus

HINARI: Health InterNetwork Access to Research Initiative

HIV: Human Immunodeficiency Virus

ICCC: Innovative Care for Chronic Conditions

ICDM: Integrated Chronic Disease management

LMICs: Low and Middle-Income Countries

MeSH: Medical subject heading

NCDs: Non-Communicable Diseases

PACTR: Pan African Clinical Trials Registry

PHC: Primary Health Care

PICO: Population, Intervention, Comparator and Outcome

PRISMA: Preferred Reporting Items for Systematic review and Meta-Analysis

QCTs: Quasi-randomised Controlled Trials

RCTs: Randomised Controlled Trial

ROBINS-I: Risk Of Bias In Non-randomized Studies – of Interventions

SE: Standard Error

T2DM: Type 2 Diabetes Mellitus.
